# BMI, physical inactivity, cigarette and alcohol consumption in female nursing students: a 5-year comparison

**DOI:** 10.1186/1472-6920-14-82

**Published:** 2014-04-17

**Authors:** Franziska Lehmann, Katharina von Lindeman, Jörg Klewer, Joachim Kugler

**Affiliations:** 1 Department of Public Health, Dresden Medical School, Dresden University of Technology, Dresden, Germany; 2 Department of Public Health and Health Care Management, University of Applied Sciences Zwickau, Zwickau, Germany

**Keywords:** Nursing students, Health promotion, Alcohol, Cigarettes, Body mass index, Physical activity

## Abstract

**Background:**

Nursing staff are often involved in counseling patients with regard to health behavior. Although care promoting healthy lifestyle choices is included in the curriculum of nursing students in Germany, several studies of nursing students have reported a high prevalence of unhealthy behavior. This paper focuses on the behavior of female nursing students with regard to body mass index (BMI), physical activity, and cigarette and alcohol consumption. It describes trends through the comparison of results from 2008 and 2013.

**Methods:**

Data was collected in two waves at a regional medical training college. First, 301 nursing students were asked to fill out a 12 page questionnaire on health behavior in 2008. The questioning was repeated in 2013 with 316 participating nursing students using the previous questionnaire.

**Results:**

259 female nursing students completed the questionnaire in 2013. 31.6% of them were either overweight or obese, 28.5% exercised less than once a week, 42.9% smoked between 10 and 20 cigarettes a day and 72.6% drank alcohol, wherefrom 19.7% consumed alcohol in risky quantities. In comparison to the data of 266 female nursing students from 2008, there were significant differences in the BMI and alcohol consumption: The percentage of overweight and obese students and the percentage of alcohol consumers at risk increased significantly.

**Conclusions:**

Health behavior of female nursing students is often inadequate especially in regard to weight and cigarette and alcohol consumption. Strategies are required to promote healthy lifestyle choices.

## Background

Body weight, physical inactivity and cigarette and alcohol consumption are important health-related lifestyle factors affecting the global burden of disease [1]. In adults the highest per capita consumption levels of alcohol can be found in several countries of the Northern Hemisphere, including Germany [2]. The consequences of hazardous health behavior are extensive on individual and social level. In Germany costs due to alcohol related diseases are estimated to exceed 27 billion Euro [3]. A health-economical estimation claimed that 3.3% of total healthcare expenditures in Germany can be attributed to smoking [4]. The numbers of smokers, drinkers and overweight is high. Results of a representative survey detected 25% women and 40% men, who consumed alcohol at-risk and a share of 30% daily smokers [5,6]. According to epidemiological studies, more than half of the German population is overweight with a body mass index (BMI) over 25 [7].

For health care professionals in particular, it is necessary to identify with a healthy lifestyle in order to serve as a role model for patients [8]. Nursing staff are often involved in counseling patients with regard to appropriate health-related behavior [9]. Although care that promotes healthy choices is included in the curriculum of nursing students in Germany, several studies of nursing students have reported a high prevalence of unhealthy behavior among the students questioned [10-12]. A survey at a regional college for health care professions in 2008 showed that overweight, physical inactivity and smoking and alcohol consumption were quite common. Approximately 40% of the surveyed nursing students were smokers, 40% alcohol consumers and 20% were overweight or obese [13,14].

The aim of this study was to repeat the survey at the same nursing college in order to compare the findings with regard to body weight, physical inactivity, and cigarette and alcohol consumption with the survey results from 2008.

## Methods

At a regional college for health care professions in Saxony, this survey was conducted as part of a series of lectures on health sciences, approved by the school committee. The questioning took place at two periods of time. Initially, 301 nursing students were surveyed in 2008 within a 90 min lecture. In the year 2013, the questioning was repeated with 316 nursing students using the same questionnaire. Participation was both voluntary and anonymous. All data were analyzed in compliance with the Helsinki Declaration.

### Questionnaire

The questionnaire included questions about the participant’s socio-demographical background, weight, diet, physical activities and smoking and drinking habits. This instrument had already been used in studies with other health care professionals and medical students [15-17].

The BMI is a commonly used index for the classification of overweight and obesity in adult individuals. It was calculated using the self reported body weight and height (kg/m^2^) as classified according to the WHO [18].

The questions relating to physical activity included, how often (less than once, once or twice, more than 3 times) and how many hours per week the respondent performed physical activities, which lasted at least 20 minutes and increased the heart and breath frequency. Predefined motives for engaging in physical activity were staying fit, knowing about the own performance status, meeting with people, balancing the educational demands, having fun, joining with family or friends, following medical advises or other reasons. Students could further state, that they do not exercise because they have not enough time, health problems, different hobbies, incompatible working schedules or due to other reasons. Multiple answers were possible.

Questions with regard to smoking habits included information about the number of smoked cigarettes, which was classified as: none, less than 10, less than 20, less than 40 or more than 40 cigarettes per day. Motives for smoking were predefined as stress or trouble, acceptance, relaxation, pastime, without reason, visits to discotheques or pubs or for other reasons. Multiple answering was allowed.

To evaluate the alcohol consumption, the alcoholic beverages beer, wine, mixed beer, alco-pops (i.e. cola-rum or vodka-lemon) and hard liquors were separately quantified in liter per week. In addition, the intake of small 2 cl liquor bottles was asked. Also it was possible to state “I don’t drink alcohol”. A multiple choice of reasons for drinking alcohol was given and included the occasion with partners, with friends or colleagues, at get-togethers or parties, in discotheques or pubs, at the weekend, without reason, after stress and trouble, daily or others. The use of alcohol was classified in two categories according to the limit of tolerable upper alcoholic quantity. An intake of pure alcohol up to 12 g a day in women and 24 g in men was classified as low-risk drinking [19]. Higher quantities of alcoholic intake were classified as at-risk drinking. Respondents who stated not to drink any alcohol were considered as abstinent. The quantity of pure alcohol in grams was calculated through the term (*x* ml * (Vol.-%/100)* 0.8) for every beverage. The average per mil was considered for beer 4.8%, mixed beer 2.5%, wine 11%, alco-pops 5.5% and hard liquors 32%.

### Statistics

The statistical analysis was done using SPSS 21.0. The data of BMI, physical activity, and cigarette and alcohol consumption from 2013 were compared with data from 2008 using the Chi-square test for homogeneity. Differences of p < 0.05 (adjusted with Bonferroni correction) were presumed to be statistically significant. The non-parametric Mann–Whitney-*U* test was used for comparisons of means, when data didn’t show normal distribution.

## Results

From a total of 420 respondents, 316 were nursing students. The survey in 2008 included 301 nursing students.

The overall response rate was 100% in 2013 and 2008. The following analysis only refers to female nursing students due to the low percentage of male respondents in 2008 and 2013 (Table 1). From the total of 259 female nursing students of the recent evaluation, 36.3% were in first year of study, 29.3% in second and 34.4% in third year of study. 90.8% of all respondents were aged between 16 and 24 years (Table 1). In 2008, 266 female nursing students participated, about one third in every year of study. 96.6% were also aged between 16 and 24 years old.

**Table 1:** Socio-demographic data of nursing students in 2008 and 2013

	**Total**	**Gender n**		**Age in years % females**			**Year of education % females**		
		**Men**	**Women**	**16 - 24**	**25 - 30**	**> 30**	**1**	**2**	**3**
**2008**	301	35	266	96.6	3.0	0.4	32.0	35.3	32.7
**2013**	316	57	259	90.8	7.7	0.8	36.3	29.3	34.4

### BMI

**Figure 1:**
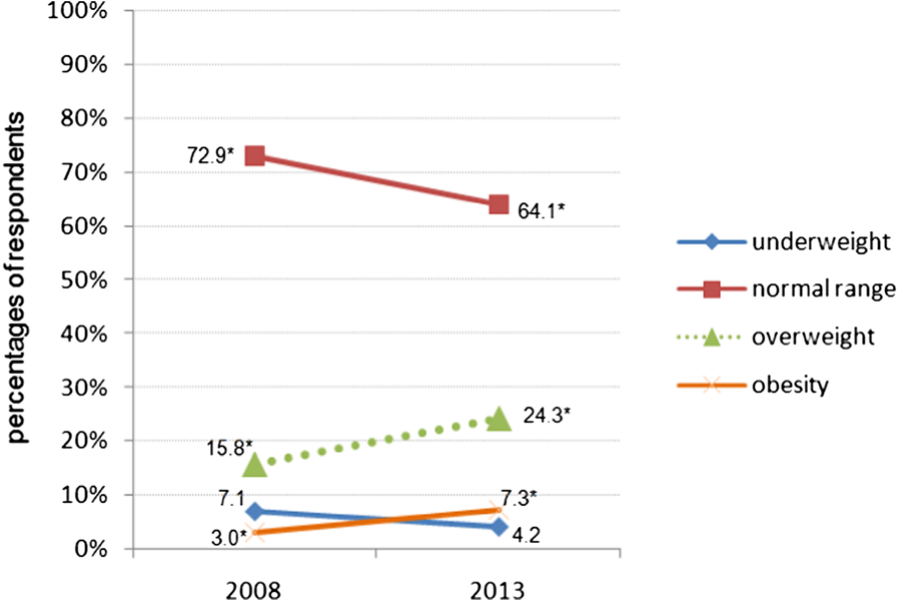
**Percentages of BMI categories indicated by BMI of female nursing students in 2008 and 2013.** 2008 (n = 266), 2013 (n = 259), *adjusted standardized residuals over ≥2 in Chi-square test.

More than half of the female nursing students had a normal weight (64.1%) and nearly a quarter of them were overweight (24.3%). Only a minority was underweight or obese (Table 2). The Chi-square test found significant differences. In comparison with the survey of 2008, the percentage of normal weight women decreased and the prevalence of overweight and obesity increased significantly (Figure 1).

**Table 2:** Health behavior in comparison between 2008 and 2013

	**n**		**%**		***p***
	**2008 2013**		**2008 2013**		
**Total**	**266**	**259**	**100**	**100**	
**BMI**					0.005*
Underweight ***<*** *18.5*	19	11	7.1	4.2	
Normal range *18.5-24.9*	194	166	72.9	64.1	
Overweight *25.5-29.9*	42	63	15.8	24.3	
Obesity *≥30.0*	8	19	3.0	7.3	
Missing	3		1.1		
**Physical activity**					0.306
0 > 1 hour/week	34	37	12.8	14.3	
1–2 hours/week	96	87	36.1	33.6	
2 < 4 hours/week	54	68	20.3	26.3	
> 4 hours/week	81	66	30.5	25.5	
Missing	1	1	0.4	0.4	
**Cigarette use**					0.274
Nonsmoker	157	148	59.0	57.1	
< 10 cig/day	83	75	31.2	29.0	
≥ 10 cig/day	25	36	9.4	13.9	
Missing	1		0.4		
**Alcohol use**					0.003*
Abstainers	58	69	21.8	26.6	
Less risk	177	137	66.5	52.9	
At- risk	30	51	11.3	19.7	
Missing	1	2	0.4	0.8	

*significance level p < 0.01 with Bonferroni correction in Chi-square test.

### Physical activity

The majority of respondents exercised regularly: on average, 3.5 h (±3.7) per week for females. In 2008, the mean time for exercising was 3.5 h (±3.2). The Mann–Whitney-*U* test was not significant (p = 0.749). A percentage of 28.6% was less than once, 52.1% once or twice and 18.9% at least 3 times a week active. The duration of physical activity differed between none or less than one hour (14.3%), one up to two hours (33.6%), two up to four hours (26.3%) and more than four hours (25.5%) (Table 2). The overall most mentioned motive for exercise was the maintenance of fitness (76.4%). Common reasons for not exercising were not enough time (32.4%) and incompatible work schedules (21.6%). Similar reasons were stated in 2008. Data comparison between 2013 and 2008 showed no significant differences in frequency or duration of physical activities.

### Cigarette consumption

With regard to smoking, 57.1% stated to be a nonsmoker: That included never smokers (48.6%), ex-smokers abstinent for at least one year (5.8%) and ex-smokers abstinent for more than one year (2.7%). Of the 42.9% smokers, 29.0% smoked less than 10 cigarettes per day and 13.9% smoked from 10 up to 20 cigarettes daily. No one stated to smoke more than 20 cigarettes a day (Table 2). Reasons for smoking were stress or trouble (35.1%), visits to discotheques or pubs (33.2%) or for relaxation (30.1%). There were no significant differences between female smokers and nonsmokers in comparison to 2008.

### Alcohol consumption

**Figure 2:**
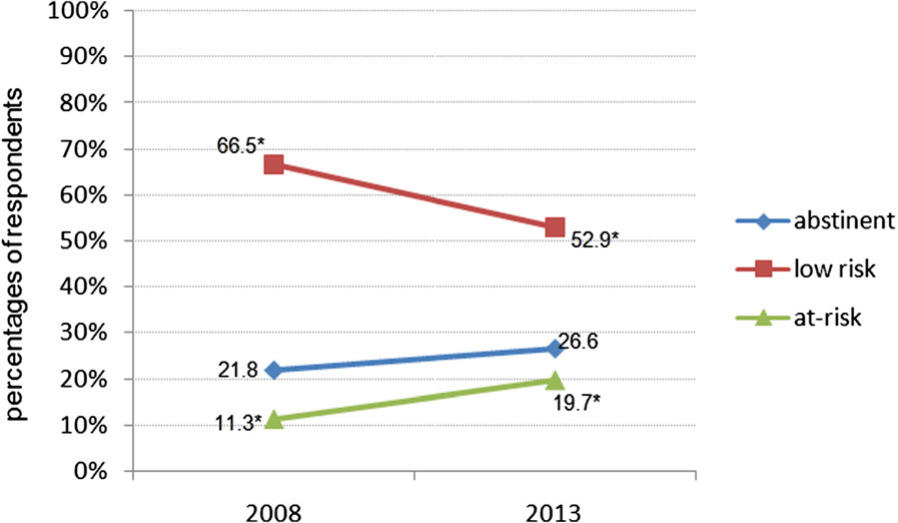
**Percentages of the categorized alcohol consumption of female nursing students in 2008 and 2013.** 2008 (n = 266), 2013 (n = 259), *adjusted standardized residuals over ≥2 in Chi-square test.

The population of female nursing students could be divided into 26.6% abstainers, 52.9% low-risk consumers and 19.7% at-risk consumers (Table 2). The mean of alcoholic consumption showed a value of 8.75 g (±20.25) daily. Overall reasons for drinking alcohol were get-togethers or parties (83.8%) and visits to discotheques or pubs (66.0%). No one stated to drink alcohol daily. Only few students drank alcohol because of stress or troubles (4.2%) or without any reason (4.2%). Alcohol consumption was significantly higher compared to data from 2008. While the percentage of abstainers didn’t differ much, more students drank at high risk (Figure 2). In addition, the mean quantity of alcohol intake was significantly higher in 2013 than in 2008: 8.75 g/d vs. 4.80 g/d (Mann–Whitney-*U*-test; p < 0.0001).

## Discussion

Health behavior of nursing students is often inadequate in terms of dietary, physical activity, smoking and alcohol drinking habits. The results concerning students BMI correspond with studies of the German general population, where 68.2% of adults aged 18 up to 29 years had normal ranged weight [7]. However, the prevalence of overweight female nursing students (24.3%) exceeded that of females of the general population (17.2%). The prevalence of smokers and never smokers were comparable to findings of a representative health study in Germany (DEGS). It identified a prevalence of 40% smokers and 45.5% never smokers among women aged between 18 and 29 years [5].

Results of DEGS stated additionally, that at-risk drinking is most common in adults aged between 19 and 29 years old, which represent the surveyed study population [6]. Alcohol consumption correspondents with findings of the national drug affinity study [20]. However, the increase of at-risk alcohol consumption within the population of female nursing students between 2008 and 2013 is concerning. The alcohol intake was on average about 9 g per day which is nearly twice as high compared with 2008. Moreover, it is twice as high as the average alcohol consumption of 16 and 17 years old girls of German general population [21].

All these points of inadequate health behavior do not reflect the requirements of health promotion and seems somewhat contradictory to successful health care education. These students will play an important role model in primary health care settings having a great potential to promote healthy choices through direct contact to patients [9]. Practicing a healthy lifestyle seems to be related with counseling and screening of patients: The Women Physicians’ Health Study reported that physicians were more likely to screen their patients for cholesterol or counsel for tobacco cessation when eating less fat or being nonsmokers themselves [8]. In addition, health professionals are considered more motivating and authentic when they demonstrate a healthy lifestyle themselves [22]. Counseling therefore is an effective treatment strategy against tobacco use. It leads to increasing abstinence among adolescent smokers [23]. The question remains wether the students are adequately informed about their ability to influence patients and if they estimate correctly their self efficacy. A different approach in lecturing seems to be required to improve consequences of health promotion curriculum to counseling skills and health behavior [24,25]. An evaluation of an online tobacco cessation course demonstrates that the individual ability and skill in counseling patients improves outcomes significantly [26].

Nationwide surveys indicated a decreasing trend in the prevalence of smoking in young adults: 43.1% in 2008 vs. 36.8% in 2011 [20]. In the present 5-year comparison, this trend could not be found in female nursing students. Similarly, nationwide surveys indicated a decreasing trend in risky alcohol consumption in young adults. Quite the contrary shows this 5-year comparison: more female nursing students tend to drink alcohol on a risky level.

The World Health Organization recommends exercising moderately for at least 150 minutes or to exercise intensively for at least 75 minutes per week. [27]. Nationwide surveys showed that 30.2% of young adults meet this recommendation [28]. The findings of this study confirm a comparable percentage of female nursing students fulfilling this recommendation. Given the increasing prevalence of obesity between 2008 and 2013 but constantly high intensity of practiced physical activities, further investigation should focus on dietary habits [29].

Most of the nursing students were female in both surveys, what is representative for that setting. The Global Health Professions Student Survey showed similar gender differences in nursing education sites all over the world with an average of 70% females [30].

This survey has some limitations. First, the results may have just restricted validity due to estimation per self report. Studies showed, that the correlation between self reported and objective measures of body weight, height and physical activity is low and varies individually [31,32]. Meanwhile the estimation of smoked cigarettes in self reports is mostly correct the validation of self reported consumption of alcoholic beverages in liter still has to be performed [33]. Even though, the use of questionnaires for assessing health behavior is a well established method [34]. It leads to high response rates, like achieved in this survey with hundred percent return, and provides numerous information in short time.

Another limitation is the missing analysis of possible relationships between smoking, alcohol and weight status. In the evaluation of the data from 2008 a cluster-analysis had been integrated by Lindeman et al. [13]. The results show a share of 35.7% smokers in the alcohol high-risk group. Similar group specificities may exist within the data 2013 and should be investigated in further analysis.

## Conclusion

If health care professionals are regarded as potential health promoters, then their own health behavior represents an important key function. Results of this survey confirm the findings of the former survey from 2008. Being overweight, smoking and alcohol drinking at risk are quite common characteristics of the health behavior of nursing students during their time of study. Further investigations should focus on determinants for deficiencies and clarify opportunities for improving the transfer of health promotion theory from the classroom into the daily lifestyle of nursing students.

## Competing interests

The authors declare that they don’t have competing interests.

## Authors’ contributions

FL performed the data acquisition, the statistical analysis and the manuscript draft. KvL managed the questioning 2013 and provided the dataset from the questioning 2008. JKl was significantly involved in developing the questionnaire and adjusted the content through integrating new items. JKu developed the questionnaire and supported the thematic focus of the analysis. All authors read and approved the final manuscript.

## Pre-publication history

The pre-publication history for this paper can be accessed here:

http://www.biomedcentral.com/1472-6920/14/82/prepub
